# Stapled Anoplin as an Antibacterial Agent

**DOI:** 10.3389/fmicb.2021.772038

**Published:** 2021-12-13

**Authors:** Monika Wojciechowska, Julia Macyszyn, Joanna Miszkiewicz, Renata Grzela, Joanna Trylska

**Affiliations:** ^1^Centre of New Technologies, University of Warsaw, Warsaw, Poland; ^2^College of Inter-Faculty Individual Studies in Mathematics and Natural Sciences, University of Warsaw, Warsaw, Poland; ^3^Division of Biophysics, Institute of Experimental Physics, Faculty of Physics, University of Warsaw, Warsaw, Poland

**Keywords:** multidrug resistance bacteria, antibacterial peptides, anoplin, stapled peptides, stapled anoplin, amphipathic helix, hydrocarbon stapling

## Abstract

Anoplin is a linear 10-amino acid amphipathic peptide (Gly-Leu-Leu-Lys-Arg-Ile-Lys-Thr-Leu-Leu-*NH_2_*) derived from the venom sac of the solitary wasp. It has broad antimicrobial activity, including an antibacterial one. However, the inhibition of bacterial growth requires several dozen micromolar concentrations of this peptide. Anoplin is positively charged and directly interacts with anionic biological membranes forming an α-helix that disrupts the lipid bilayer. To improve the bactericidal properties of anoplin by stabilizing its helical structure, we designed and synthesized its analogs with hydrocarbon staples. The staple was introduced at two locations resulting in different charges and amphipathicity of the analogs. Circular dichroism studies showed that all modified anoplins adopted an α-helical conformation, both in the buffer and in the presence of membrane mimics. As the helicity of the stapled anoplins increased, their stability in trypsin solution improved. Using the propidium iodide uptake assay in *Escherichia coli* and *Staphylococcus aureus*, we confirmed the bacterial membrane disruption by the stapled anoplins. Next, we tested the antimicrobial activity of peptides on a range of Gram-negative and Gram-positive bacteria. Finally, we evaluated peptide hemolytic activity on sheep erythrocytes and cytotoxicity on human embryonic kidney 293 cells. All analogs showed higher antimicrobial activity than unmodified anoplin. Depending on the position of the staple, the peptides were more effective either against Gram-negative or Gram-positive bacteria. Anoplin[5-9], with a lower positive charge and increased hydrophobicity, had higher activity against Gram-positive bacteria but also showed hemolytic and destructive effects on eukaryotic cells. Contrary, anoplin[2-6] with a similar charge and amphipathicity as natural anoplin effectively killed Gram-negative bacteria, also pathogenic drug-resistant strains, without being hemolytic and toxic to eukaryotic cells. Our results showed that anoplin charge, amphipathicity, and location of hydrophobic residues affect the peptide destructive activity on the cell wall, and thus, its antibacterial activity. This means that by manipulating the charge and position of the staple in the sequence, one can manipulate the antimicrobial activity.

## Introduction

The problem of bacteria gaining resistance to existing antibiotics is constantly growing. Every year, the WHO reviews the research on the development of new antimicrobial agents. Last year’s report (WHO, 2020) about traditional and non-traditional antibacterials in preclinical and clinical phases points out the dissatisfying number of antimicrobials in the clinical trials. Just 43 agents based on traditional antibiotics and 27 based on non-traditional antibacterial entities have reached clinical phases. Importantly, only two are active against multidrug resistance (MDR) Gram-negative bacteria. Due to the increased presence of MDR bacteria, there is still a substantial demand for new effective antibiotics. For the first time, WHO reviewed non-traditional agents with antibacterial potential. New innovative non-traditional antibacterial agents include bacteriophages, virulence inhibitors, antimicrobial peptides (AMP), immunomodulatory compounds, and vaccines. Over 10% of all potential antimicrobials in the preclinical research phase are AMP (WHO, 2020).

Plants and animals evolutionary developed several defense mechanisms against pathogens. AMP are one of many components of the natural immunity systems of live organisms. These peptides show a broad spectrum of activity against Gram-positive and Gram-negative bacteria, fungi, viruses, and parasites (Hancock and Diamond, 2000). AMP have increasingly been explored as an alternative to conventional antibiotics (Yan et al., 2021). Currently, the sequences of approximately 3,200 AMP are known (Wang et al., 2016). The majority of AMP has similar characteristics: small size, cationic charge, and amphipathic nature. The simplest classification of AMP is based on their structure and includes linear, α-helical, antiparallel β-sheets stabilized by intramolecular disulfide bridges, combined α/β, and cyclic structures (Huan et al., 2020; Li et al., 2021b). In addition, many AMP with more complex topologies have been reported, especially among peptides from plant sources (e.g., defensins, cyclotides, thionins, knotting-type, and hevein-like peptides; Ribeiro et al., 2013; Koehbach and Craik, 2019; Li et al., 2021a). The mechanism of AMP action mostly involves their interaction with the bacterial cell membrane and damage of the phospholipid bilayer, which causes the loss of membrane continuity. This process is closely related to the cationic charge and structure of the peptides (Datta and Roy, 2021). The moderate clinical success of AMP is associated with the general limitations of natural cationic peptides. The main disadvantages of AMP are toxicity, possible immunogenicity, and protease sensitivity (Lei et al., 2019; Huan et al., 2020). Hence, new chemical modifications of peptides should be developed to create protease-resistant, less toxic, and more antibacterially active peptides (Yan et al., 2021; Li et al., 2021b).

Our interests focused on the peptides that primarily take on an active structure only near the lipid environment and close to the bacterial cells (Wojciechowska et al., 2020). A promising approach to improve the pharmacological properties of AMP may be stabilizing their active conformation. A typical membrane-active AMP conformation is an α-helix (Lei et al., 2019). One of the increasingly studied ways to stabilize the active α-helical structure is by introducing a hydrocarbon staple into a peptide sequence (Sahariah et al., 2015; Ali et al., 2019). Several recent reports demonstrated that a stapled α-helix can serve to develop short amphipathic AMP that showed high proteolytic stability and low toxicity (Jenner et al., 2017; Luong et al., 2017b; Stone et al., 2018; Mourtada et al., 2019).

Anoplin is a short, 10-amino acid peptide of natural origin with a broad spectrum of biological activities, including the antibacterial one (Konno et al., 2001). Many research groups investigated multiple modifications of anoplin to improve its pharmacological activity (Wu et al., 2020). Our studies showed low antibacterial activity of this peptide against *Escherichia coli* strains and confirmed that anoplin adopts an α-helical conformation only near the lipid environments (Wojciechowska et al., 2020). Therefore, we hypothesized that stabilizing the α-helix of anoplin would improve its antibacterial activity. In this work, we investigated the effect of hydrocarbon stapling of anoplin on its antimicrobial activity. Firstly, we designed and synthesized selected stapled anoplin sequences and examined their antimicrobial efficacy for a range of Gram-negative and Gram-positive bacteria. Secondly, using circular dichroism (CD) spectroscopy, we investigated the propensity to form the helical structure by the stapled anoplin in different membrane environments. Thirdly, we investigated the proteolytic stability of these peptides in trypsin solution. Next, we evaluated the disruption efficiency of bacterial membranes after incubating bacteria with the peptides and propidium iodide (PI) solution. Finally, we examined the cytotoxicity of anoplin and its analogs against human embryonic kidney 293 cells (HEK 293) and hemolytic activity on sheep red blood cells (RBC). This is the first work that demonstrates the potential of a stapled anoplin as an effective antibacterial including a comprehensive study of the effects of such modifications.

## Materials and Methods

The sodium dodecyl sulfate (SDS), lipopolysaccharides (LPS), 2-Oleoyl-1-palmitoyl-sn-glycero-3-phosphocholine (POPC), 2-Oleoyl-1-palmitoyl-sn-glycero-3-phospho-rac-1-glycerol (POPG), and 2-Oleoyl-1-palmitoyl-sn-glycero-3-phosphoethanolamine (POPE) lipids, the resin for peptide synthesis, and protected amino acids were obtained from Sigma-Aldrich. Sheep RCB were from GRASO (Poland). All chemicals were of analytical or reagent grade and the buffers were prepared using distilled water.

### Synthesis of Stapled Anoplins

All peptides were synthesized manually with the Fmoc chemistry solid-phase peptide synthesis (Stawikowski and Fields, 2002) on a Rink-amide resin (Tenta Gel S RAM, amine groups loading of 0.24 mmol/g; this resin has a linker which yields a C-terminal amine upon trifluoroacetic acid cleavage of the peptide). For optimal reaction yields, peptides were synthesized using two different methods of activation of amino acids. Coupling of unmodified anoplin was carried out with 3-fold molar excess of Fmoc-protected amino acids using a 3-fold molar excess of activator reagents: *O*-(7-Aza-1*H*-benzotriazole-1-yl)-*N*,*N*,*N*′,*N*′-tetramethyluronium hexafluorophosphate (HATU) and 1-hydroxy-7-azabenzotriazole (HOAt), a 6-fold molar excess of collidine and *N*,*N*-Dimethylpyridin-4-amine as a catalyst, whole dissolved in the dimethylformamide/*N*-methylpyrrolidone (DMF/NMP; 1:1,v:v) as a solution and mixed for 1.5 h. For peptides containing (S)-2-(4′-pentenyl)-alanine (S_5_) in the sequence, a different amino acid activation method was used: 4-fold molar excess of Fmoc-protected amino acids, HATU, HOAt, and 8-fold molar excess of *N*-Ethyldiisopropylamine (DIPEA). All acylation processes were carried out two times for 45 min, except for amino acids following the unnatural S_5_ residue. In this case, the reaction was repeated three times. The Fmoc deprotection was accomplished using 20% piperidine in DMF for 2 cycles through 10 min for natural amino acids, whereas for the S_5_ residues – 4 cycles for 10 min (Bird et al., 2011). Ring-closing metathesis (RCM) was performed using a 0.25-fold molar excess of the first Generation of Grubbs Catalyst dissolved in degassed dichloroethane (DCE). The solution and resin were stirred at room temperature for 2 h under the nitrogen atmosphere (Chapuis et al., 2012). The metathesis reaction was repeated three times with a fresh portion of Grubbs catalyst to complete the reaction. Finally, the Fmoc protecting group was removed from the last N-terminal amino acid after RCM reaction. The peptide was deprotected and cleaved from the resin by treatment with a trifluoroacetic acid/triisopropylsilane/water (95:2.5:2.5,v/v/v) mixture for 3 h.

The synthesized peptides were analyzed and purified by reverse phase high-performance liquid chromatography (RP-HPLC) analytical and semi-preparative (Knauer C18 columns, 5 μm particles, 4,6 × 250 mm, and 8 × 250 mm, respectively). Peptides were purified in different mobile phase gradients with buffer A (0.1% trifluoroacetic acid in acetonitrile) and buffer B (0.1% trifluoroacetic acid in water) at a flow rate of 1.5 ml/min and wavelength 220 nm (from 20 to 80% for 30 min for anoplin, from 30 to 70% for 30 min for anoplin[2-6], from 30 to 60% for 30 min for anoplin[5-9], and from 40 to 60% for 30 min for anoplinS_5_(5,9)). The purity and identity of peptides were checked by RP-HPLC and mass spectrometry using the Q-TOF Premier mass spectrometer (see Supplementary Figures 1–4). To exchange the anion to hydrochloride before the spectral measurements, peptides were dissolved in a 0.1 M HCl solution, frozen, and lyophilized.

### Recording of CD Spectra

CD spectra were recorded in aqueous buffer solution (10 mM phosphate buffer, pH 7.0), in the presence of 5 mM SDS, 2 mM dodecylphosphocholine (DPC), 50 μM LPS micelles, and 1 mM POPC:POPG (3:1) or 1 mM POPC:POPE (3,1) phospholipids, as described in Wojciechowska et al. (2020). In all CD experiments, the peptide concentrations were 120 μM. The spectra were collected using the Biokine MOS-450/AF-CD spectrometer equipped with the Xe lamp using a 0.1 cm CD cell. The acquisition duration time was 2 s with a resolution of 1 nm. The measurements were performed in 10 mM phosphate buffer, pH 7.0, room temperature, and wavelength range 190–260 nm. The CD spectra were smoothed with the Savitzky-Golay method and presented using GraphPad. Contributions from micelles and small unilamellar vesicles (SUVs) were eliminated by subtracting their spectra from the corresponding peptide with micelles and SUVs. For peptides in SUVs, the CD data below 190 nm were not taken for analysis because their recording produced a high-tension value over 600 V giving a low signal-to-noise ratio. The presented CD spectra are the average of three scans. Every CD experiment was conducted twice. The percentage of helix in CD spectra was estimated based on the DichroWeb software (Whitmore and Wallace, 2004). The spectra were analyzed with the CDSSTR analysis program and DataSet4 data set (Whitmore and Wallace, 2008; Abdul-Gader et al., 2011).

### Trypsin Digestion Assay

250 μl of peptide solution in ammonium bicarbonate buffer (0.1 M NH_4_HCO_3_, pH 8.0) and an appropriate amount of trypsin (Sigma-Aldrich) solution in water were incubated at 37°C with rapid shaking (600 rpm). Trypsin and peptides were mixed in the ratio (1:1333; Luong et al., 2017b). After 2, 4, 6, and 8 min of incubation, 60 μl of digestion mixture was taken and treated with 60 μl of a solution of 50% acetonitrile containing 1% TFA to stop the digestion. Peptide degradation was analyzed with RP-HPLC using analytical methods to assess peptide purity (see subsection Synthesis of stapled anoplins). The results were calculated as the percentage of peptide peak of area degradation. The total area of every peak was designated for experiments without trypsin. Each of the experiments was performed thrice.

### Propidium Iodide Uptake Assay

The bacterial membrane permeabilization induced by the synthesized peptides was determined using propidium iodide (PI). The experiments were performed on *E. coli* K12 and *Staphylococcus aureus* ATCC 29213. Bacteria were grown in Mueller Hinton Broth (MHB) until the optical density at 600 nm reached 0.27. Different concentrations of peptides (64, 32, 16, 8, 4, and 2 μM) were added to the wells of a black-walled microplate. 50 μl of the bacterial suspension containing PI (final concentration, 10 μM) was added to the wells. Bacteria in MHB without peptides were used as a negative control. The emitted fluorescence was measured at excitation and emission wavelengths of 580 and 610 nm, respectively, at 37°C for 2 h using a Microplate Reader Biotek (Winooski, VT, United States). Data were collected by the scan per each 1 min. The percentage of membrane permeabilization was calculated as the ratio of fluorescent intensity of the peptide treated with PI and untreated samples. The experiment was performed in triplicates.

### Bacterial Growth Inhibition

The minimum inhibitory concentrations (MICs) of anoplin, its analogs (anoplin[2-6], anoplin[5-9], anoplinS_5_(5,9)), and antibiotics were determined using the broth microdilution protocol of Clinical and Laboratory Standards method M07-A10 (CLSI, 2015). Bacteria were grown in lysogeny broth with agar (LA), then in lysogeny broth (LB) medium at 37°C with shaking. Next, to check the susceptibility to the above agents, bacteria were cultured in a cation-adjusted MHB at 37°C with shaking to the exponential phase and diluted to ~5 × 10^5^ CFU/ml. Suspended cells were added to the sterile 96-well plates containing peptides and antibiotics in different concentrations (64, 32, 16, 8, 4, 2, 1, 0.5, 0.25, and 0.125 μM). Then, the plates were incubated at 37°C with shaking for 19 h, and additionally 1 h with resazurin (Sigma-Aldrich; 0.2 mg/ml, 20 μl). The MIC values were determined in two ways: by observing resazurin color changes (blue – dead bacteria and pink – live bacteria; Sarker et al., 2007; Hafez et al., 2017) and by measuring optical density at 600 nm (OD_600_). Every MIC experiment was performed in at least three independent biological replicates for the peptides (and one for control antibiotics).

### Cytotoxicity Assay

The HEK 293 cells were seeded at a density 2 × 10^4^ per well on a 96-well plate and incubated in high glucose DMEM medium (Lonza) supplemented with 10% FBS and 50 U/ml of penicillin–streptomycin at 37°C in 5% CO_2_. After 24 h, cells were covered with a fresh medium containing tested peptides at different concentrations (4, 8, 16, and 32 μM). After 24 or 48 h of incubation, cells viability was tested using MTT [3-(4,5-dimethylthiazol-2-yl)-2,5-diphenyltetrazolium bromide] (Merck). Briefly, 10 μl of 5 mg/ml MTT was added to each well and incubated for 4 h. Formazan produced from MTT was solubilized during overnight incubation in 100 μl of 10% SDS in 0.01 M HCl. Absorbance at 590 nm was measured using Microplate Reader Biotek (Winooski, VT, United States). The experiment was performed in triplicates.

### DAPI Staining

1 × 10^5^ cells were seeded into coverslips and incubated for 24 h. The cells were then treated with anoplin (64 μM) or its derivatives (8–32 μM) for 24 h. Next, cells were washed with PBS and fixed in 4% PFA for 15 min at room temperature. After three washes with PBS, cells were stained with DAPI (4′,6-diamidino-2-phenylindole, dihydrochloride; Boster) for 5 min (as indicated in the instruction). Coverslips with cells were washed three times and mounted on a glass microscope slide using a Dako Fluorescence Mounting Medium. Images were acquired with Axio Imager Z2 LSM 700 Zeiss confocal microscope.

### Hemolysis Assay

Sheep RBC (200 μl) were pelleted (3,500 rpm for 5 min), washed three times with PBS buffer (PBS 10 mM and 150 mM NaCl, pH 7.4). The cells were then diluted in PBS buffer (10 ml), divided into 50 parts into 1.5 ml tubes, and pelleted by centrifugation. Various concentrations of peptide solutions (200 μl) were added to the cells and incubated with shaking (165 rpm, 37°C) for 30 min. The cells were pelleted by centrifugation (3,500 rpm for 5 min). Then, 100 μl of supernatant from each tube was collected into a clear 96-well plate, and the absorbance of the released hemoglobin was measured with a plate reader spectrophotometer (Microplate Reader Biotek, Winooski, VT, United States) at 405 nm. Hemolysis was determined relative to the negative control (PBS) and the positive control (1% v/v Triton-X-100 that lyses 100% of RBC). The percentage of hemolysis was determined using the following equation:


$$\%\;\mathrm{hemolysis}=\frac{\left(A-A_{0}\right)}{\left(A_{100}-A_{0}\right)}\cdot 100\%$$

where A0 is the absorbance intensity of the RBC in buffer (background), and A and A100 are the absorbance intensities of the RBS in the presence of peptides and Triton X-100, respectively.The tests were performed with duplicate samples, and the average values of three independent measurements were recorded.

### Statistical Analyses

All data were analyzed using GraphPad Prism 7 (GraphPad Software, San Diego California, United States) statistical analysis software. Two or multiple groups of data were compared using a two-way ANOVA test. A value of *p* < 0.05 was considered to be statistically significant. The figures of peptide structures were prepared with VMD (Humphrey et al., 1996).

## Results

### Design and Synthesis of Stapled Anoplins

**Figure 1:**
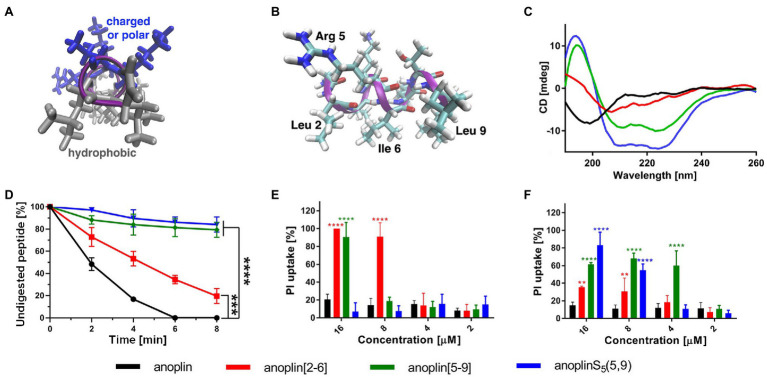
**(A)** The model of the helical structure of anoplin. The helix-forming backbone is marked in magenta. The helical-wheel view from the C-terminus showing the amphipathic character of anoplin. **(B)** The side view of anoplin denoting the residues that were substituted with staples. **(C)** CD spectra of anoplin and its analogs in the phosphate buffer (10 mM, pH 7.0). **(D)** Digestion of the peptides by trypsin as a function of time. The remaining peptide amounts were determined by RP-HPLC. **(E)** Permeabilization assay of *E. coli* K12 MG1655 and **(F)** *S. aureus* ATCC 29213 using propidium iodide (PI) indicated as the percentage of PI uptake after 30 min of incubation. Error bars represent the standard error of the mean; *n* = 3. Statistical significance between the unmodified anoplin and the modified peptides is denoted by ^****^p < 0.0001, ^***^p < 0.001, and ^**^p < 0.01. Otherwise, the differences are not statistically significant.

Typically, a staple is introduced into a peptide by inserting two unnatural amino acids with olefinic side chains: (S)-2-(4′-pentenyl)-alanine (S_5_) at sites separated by one helical turn (*x* and *x* + 4, where *x* is the position of the unnatural amino acid in the peptide sequence; Chapuis et al., 2012; Cromm et al., 2015; Ali et al., 2019). One of the challenges to overcome while designing active stapled anoplins is the selection of amino acids in the anoplin sequence to be substituted with S_5_ to further generate the hydrocarbon staple. The staple location sites were selected to reduce toxicity and improve the antibacterial activity of anoplin but at the same time to keep its amphipathic character (Figure 1A). Previous research showed that substituting anoplin positively charged amino acids (Lys and Arg) with staples increased its oncological activity in cellular models and mouse tumor models (Wu et al., 2021). Thus, we expected that Lys and Arg modifications would increase anoplin toxicity against eukaryotic cells. Therefore, we selected the uncharged amino acids, Leu at position 2, and Ile at position 6 for staple insertion (anoplin[2-6]; see Figure 1B and Table 1). However, the alanine scan of anoplin showed that the highest antibacterial activity was obtained for the derivative that contains Ala instead of Arg at position 5 (Ifrah et al., 2005). Therefore, we also introduced a staple in the Arg 5 and Leu 9 positions (anoplin[5-9]) even though it changed the anoplin total charge (see Figure 1B and Table 1). Using this approach, we compared two stapled anoplins with unmodified charge and with the net charge 1e lower as compared to the natural anoplin. Further, to test the effect of incorporation of olefinic side chains and peptide charge on anoplin activity, we synthesized anoplin substituted with S_5_ at positions 5 and 9 but not ring-closing (anoplinS_5_(5,9)). According to the guidelines in the works (Konno et al., 2001; Cabrera et al., 2008), all synthesized anoplin derivatives and unmodified anoplin contained an amide at the C-terminus. The sequences and physicochemical properties of synthesized peptides are shown in Table 1.

**Table 1:** Peptide sequence modifications form, net charge at pH 7.4, percentage of helicity calculated from the CD spectra of peptides in the phosphate buffer, determined using the DichroWeb software (Whitmore and Wallace, 2004); all peptides have an amide at the C-terminus.

Peptide name	Sequence (N->C)	Form	Net charge (e)	Helicity (%)
anoplin	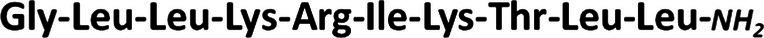	linear	+4	14
anoplin[2-6]	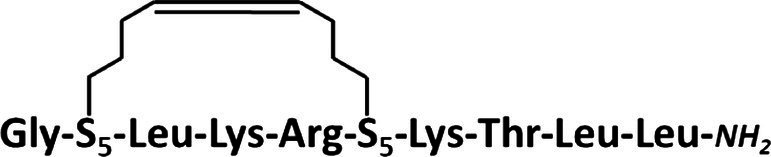	stapled	+4	59
anoplin[5-9]	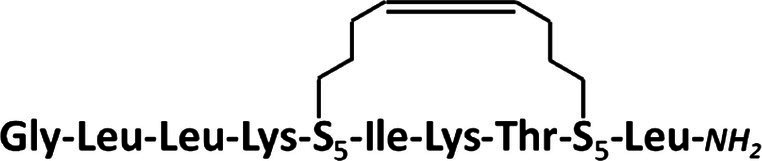	stapled	+3	64
anoplinS_5_(5,9)	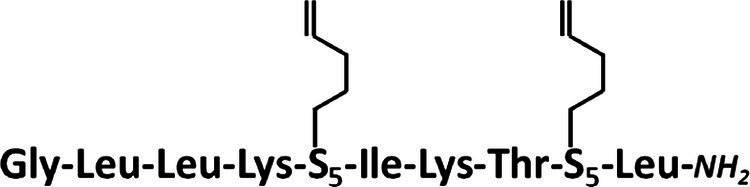	linear	+3	62

Residues S_5_ were incorporated into the peptides during solid-phase synthesis. Olefinic groups S_5_/S_5_ were cross-linked by ring-closing metathesis before cleaving the peptides from the resin (Chapuis et al., 2012; Dinh et al., 2014). Data confirming the presence and purity of the synthesized peptides are shown in Supplementary Figures 1–4.

### Secondary Structure Assessment

**Figure 2:**
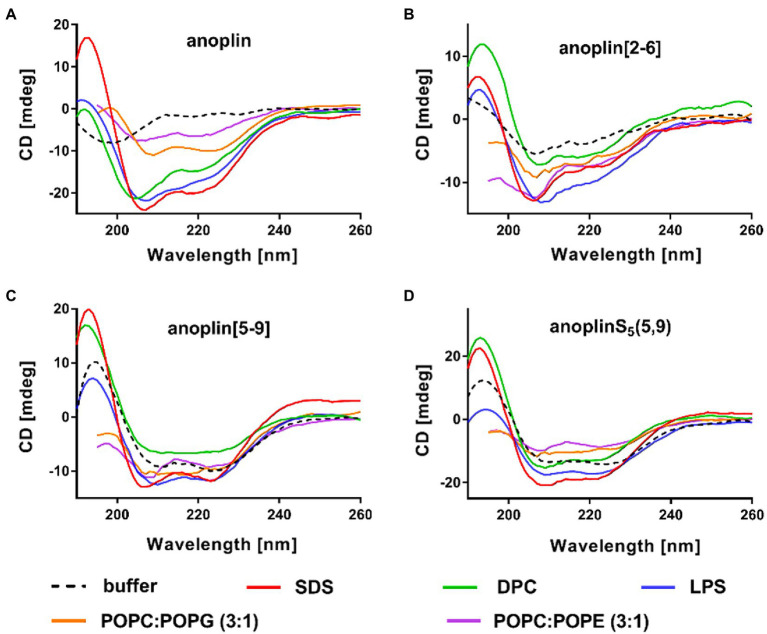
CD spectra of **(A)** anoplin, **(B)** anoplin[2-6], **(C)** anoplin [5-9], and **(D)** anoplinS_5_(5,9) in different membrane environments: phosphate buffer, SDS, DPC micelles, POPC:POPG (3:1) SUVs, POPC:POPE (3:1) SUVs, and LPS.

CD was used to determine the secondary structure of the peptides in the buffer solution (Figure 1C) and the environments mimicking membranes (Figure 2). As shown earlier (Konno et al., 2001; Wojciechowska et al., 2020), in the phosphate buffer, anoplin adopted a random coil (Figure 1C; Table 1). As expected, the introduction of staples into the anoplin sequence induced the α-helical structure (Figure 1C red and green lines). The percentage of the helix in stapled anoplin[2-6] increased to 59% and anoplin[5-9] to 64% as compared to the unmodified peptide (Table 1). A surprisingly clear representative spectrum of an α-helix was obtained for anoplinS_5_(5,9) in the phosphate buffer (Figure 1C, blue line).

SDS and SUVs composed of POPC and POPG lipids are negatively charged, so they resemble prokaryotic membrane mimics. DPC micelles and SUVs composed of POPE and POPG lipids are neutral, so they resemble eukaryotic membrane mimics. The POPC:POPG or POPC:POPE mixtures were used in a molar ratio of 3:1 (Russell et al., 2012; Wojciechowska et al., 2020). We also investigated the change of peptide secondary structure in the presence of LPS isolated from the *E. coli* O111:B4 strain (Avitabile et al., 2015). We found that the peptides formed a helix in all these membrane-mimicking environments, which is signified by prominent minimum bands at 208 and 222 nm (Figure 2; Miles and Wallace, 2016) and percentages of helical structures quantified from the CD spectra by DichroWeb (Whitmore and Wallace, 2004, 2008; Supplementary Table 1).

### Digestion of Peptides With Trypsin

To test the proteolytic resistance of anoplin derivatives, the peptides were incubated with trypsin for indicated periods (Figure 1D). The effects of the protease digestion were determined by HPLC analyses. The unstapled anoplin was susceptible to trypsin, resulting in the complete decay of the peak from the peptide as early as 4–6 min of incubation (Figure 1D; Supplementary Figure 5). However, introducing staples into the anoplin structure increased peptide stability in the enzyme solution. The decline of the peptide peak areas that appear on chromatograms after 8 min of incubation showed statistically significant differences between the stability of anoplin and the stabilities of modified anoplins. As shown in Figure 1D, anoplin[5-9] and anoplinS_5_(5,9) exhibited significantly higher stability against trypsin than anoplin and anoplin[2-6].

### Monitoring Integrity of the Bacterial Membrane

We monitored bacterial membrane destabilization in real time using the PI uptake assay by Gram-negative *E. coli* K12 MG1655 and Gram-positive *S. aureus* ATCC 29213 strains. In general, the PI fluorescent dye does not penetrate the integral bacterial membrane. However, if the integrity of the membrane is disrupted, PI penetrates the damaged membrane and binds to DNA, increasing fluorescence (Benincasa et al., 2016; Zhong et al., 2020). Therefore, we measured the total fluorescence emitted by bacteria after incubation with different concentrations of the peptides. Figures 1E,F show the percentage of PI uptake by bacteria after 30 min incubation with anoplin and its stapled analogs.

The efficiency of permeabilization of the bacterial membranes depends on the position of the staple in the peptide sequence and type of bacteria (Figures 1E,F). In addition, the increase of the PI fluorescence depends on the peptide concentration. The cell membranes of Gram-negative bacteria were destabilized most by anoplin[2-6], already at 8 μM concentration. Other peptides at this concentration exhibited a very low PI uptake in *E. coli*. The permeabilization efficiency in Gram-positive bacteria differed, with the highest PI uptake observed for anoplin[5-9]. The differences between anoplin[5-9] and other peptides were especially pronounced for concentrations of 4 μM.

### Antimicrobial Activity

The antimicrobial activity of anoplin and its stapled analogs against Gram-negative and Gram-positive bacteria was determined as the lowest concentration inhibiting the bacterial growth (Table 2; Supplementary Figures 6–15). The parent peptide showed overall low antimicrobial activity, with the MIC between 16 and 64 μM for both Gram-negative and Gram-positive strains. Introducing olefinic side chains into the anoplin sequence slightly improved its activity but only toward the Gram-positive strains. AnoplinS_5_(5,9) still had low antimicrobial activity against Gram-negative bacteria, similar to the native anoplin. However, stapling of the olefinic side chains at positions 5 and 9 significantly lowered the MIC as compared to the unmodified anoplin. Especially meaningful differences were observed for Gram-positive bacteria. The MIC values of anoplin[5,9] for the *S. aureus* strains (also for methicillin-resistant strain) and *Bacillus subtilis* (*B. subtilis*) were 4 μM, which is at least 16 times lower than for the native anoplin. Contrary, anoplin stapled at positions 2 and 6 showed significantly increased activity against Gram-negative strains but was not active against *S. aureus*. The growth of all *E. coli* strains was inhibited by anoplin[2-6] already at the concentration of 4 μM. Indeed, the stapled anoplin[2-6] was active against pathogenic strains and *E. coli* extended-spectrum β-lactamase-producing (ESBL+) strains (Baraniak, 2002; Hrabák et al., 2009).

**Table 2:** Minimum inhibitory concentration (MIC) of anoplin analogs against different Gram-negative and Gram-positive strains.

Bacteria	MIC (μM)						
	Peptide				Antibiotic		
	anoplin	anoplinS_5_(5,9)	anoplin[5-9]	anoplin[2-6]	ampicillin	kanamycin	polymyxin B
**Gram-negative**							
*E. coli* K12 MG1655	32	64	16	4	8	8	0.25
*E. coli* O157:H7	64	32	8	4	8	2	0.5
*E. coli* 1841/06 ESBL+	64	32	8	4	≥64	≥64	0.25
*E. coli* WR 3551/98 ESBL+	64	64	16	4	≥64	≥64	0.5
*P. aeruginosa* PAO1	16	64	16	8	≥64	≥64	0.5
*P. aeruginosa* ATCC 27853	32	≥64	32	16	≥64	≥64	0.25
*S. typhimurium LT2*	64	≥64	32	8	≥64	≥64	0.5
**Gram-positive**							
*S. aureus* ATCC 29213	≥64	8	4	64	8	4	≥64
*S. aureus* ATCC BAA1720 MRSA	≥64	8	4	≥64	≥64	≥64	≥64
*B. subtilis* 168	16	8	4	2	0.125	0.25	2

In comparison with conventional antibiotics (ampicillin, kanamycin) and an antibiotic with a cyclic peptide structure (polymyxin B), anoplin[2-6] showed a stronger antimicrobial activity against almost all Gram-negative, while anoplin[5-9] against almost all Gram-positive, bacteria. Importantly, the stapled anoplins exhibited excellent antimicrobial potencies against pathogenic drug-resistant strains (*E. coli* O157:H7, *E. coli* 1841/06 ESBL+, *E. coli* WR 3551/98 ESBL+, and *S. aureus* ATCC BAA1720 MRSA) unlike the conventional antibiotics, like ampicillin and kanamycin.

### Toxicity Against Human Cells and Hemolytic Activity

**Figure 3:**
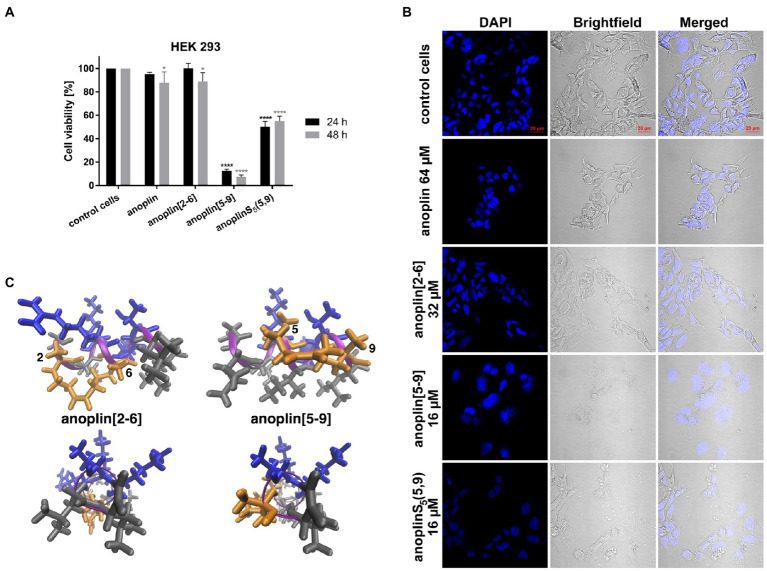
**(A)** The cytotoxic effect on HEK 293 cells of anoplin analogs at the concentration of 32 μM. The results are presented as the % cell viability in comparison with untreated control cells. Error bars represent the standard error of the mean; *n* = 3. Statistical significance between samples and untreated cells: ^****^p < 0.0001 and ^*^p < 0.05. **(B)** Confocal microscope images of the HEK 293 cells, counterstained with DAPI, untreated or incubated with indicated concentrations of anoplin, anoplin[2-6], anoplin[5-9], and anoplinS_5_(5,9). Scale bars equal to 20 μm. **(C)** The structures of stapled anoplin[2-6] and anoplin[5-9] showing the polar and non-polar residues (side and top views). Peptide main chains are indicated in pink. Hydrophobic residues are shown in gray and hydrophilic in blue. Orange represents the hydrophobic hydrocarbon staples.

Peptide cytotoxicity was tested in HEK 293 cells (Figure 3A and Supplementary Figure 16). Anoplin modified with olefinic acid at positions 5,9 displayed increased cytotoxicity against HEK 293 cells in a concentration-dependent manner. Anoplin[5-9] showed approximately 60% of dead cells at only 16 μM (Supplementary Figure 16) and the concentration of 32 μM nearly eliminated all living cells (Figure 3A). For anoplin[2-6] and unmodified anoplin, there was no significant effect on cell viability at 24 and 48 h of treatment as compared to the untreated control cells (Figure 3A; Supplementary Figure 16).

At the same time, cells treated with anoplin or its derivatives were observed using the confocal microscope (Figure 3B). The morphology of cells treated with anoplin and anoplin[2-6] was not altered even at high concentrations (64 μM of anoplin and 32 μM of anoplin[2-6]). In contrast, the altered cell appearance and the presence of cellular debris were manifested after adding anoplin[5-9] and anoplinS_5_(5,9). The unstapled peptide caused weaker effects at higher concentrations, while for the stapled one, the changes in the integrity of the nuclei were also evident.

To investigate the safety of peptides beyond cytotoxicity against the HEK 293 cells, the hemolytic activity of peptides against sheep RBC was also tested. The results are illustrated in Table 3 and Supplementary Figure 17. The results of the hemolysis experiment coincided with the cytotoxicity results on eukaryotic cells. Anoplin and anoplin[2-6] showed almost no hemolytic activity at 32 μM (only 4%), whereas anoplin [5-9] showed strong hemolytic activity (43%) at the same concentration.

**Table 3:** Hemolytic activities (%) of anoplin analogs against sheep red blood cells.

Concentration [μM]	Anoplin	Anoplin[2-6]	Anoplin[5-9]	AnoplinS_5_(5,9)
64	2.3	9.1	62.7	14.2
32	0.6	4.0	43.2	11.9
16	0.7	2.8	29.8	8.9
8	1.3	2.4	16.6	7.2
4	0.5	2.1	6.5	6.7

*Erythrocytes treated with 1% Triton-X-100 were used as positive controls and set as 100% hemolysis*.

## Discussion

We reported the antibacterial activity, cytotoxicity, hemolytic activity, proteolytic stability, bacterial membrane permeabilization efficiency, and structural changes in different membrane environments of various anoplin analogs including the stapled anoplins and one containing modified but unstapled side chains. The results confirmed that also for anoplin the stapling strategy increases the level of helicity as compared to the unmodified peptide (Chapuis et al., 2012; Luong et al., 2017a). To assess the effect of stapling on the anoplin helicity, we compared the stapled anoplin[5,9] and side chain modified but not stapled anoplinS_5_(5,9). The CD spectra in the phosphate buffer, micelles, and SUVs showed that stabilization of the helix, resulting from the incorporation of S_5_, is similar for both peptides, and independent of whether the side chains were stapled (Figure 1C; Figure 2 and Supplementary Table 1). This suggests that the introduction of these modified side chains increases anoplin propensity to form a helix. Our results corroborate observations of Chapuis et al. (2012) who showed that embedding olefinic amino acids into the peptide sequences of lasioglossum III and melectin, in some cases, increases their helical content (even if the side chains were not stapled).

We also observed a connection between the secondary structure of peptides and their stability; the higher the helix content, the higher the proteolytic resistance. Notably, the S_5_(5-9) peptide that showed a stable helix also exhibited the highest enzymatic resistance. Considering that the peptide cleavage sites by trypsin are at the C-terminal sides of Lys and Arg in the sequence, we concluded that the replacement of Arg at position 5 with S_5_ was crucial in increasing the proteolytic stability of anoplin (Manea et al., 2007).

The activity of anoplin against various pathogens was documented in many studies (Wu et al., 2020). Anoplin MIC values obtained by us against *E. coli* and *S. aureus* strains were similar to the previously reported ones (Wang et al., 2014; Libardo et al., 2015; Wojciechowska et al., 2020; Zhong et al., 2020) and are on order of tens of μM. As shown in Table 2 and Supplementary Figures 6–15, anoplin analogs were more active (with lower MIC values) than the native anoplin against all bacterial strains. Overall, anoplin[2-6] was the most active against Gram-negative, while anoplin[5-9] against Gram-positive bacteria. The only exception was that anoplin[2-6] exhibited a low MIC (of 2 μM) for one of the Gram-positive bacteria – *B. subtilis* 168. This difference is likely related to the cell wall structure. Anoplin analogs are cationic and will readily interact with anionic components of bacterial membranes. The diversity of the negatively charged membrane components of individual strains may impact the efficiency of the interaction of the peptides with membranes. The main target of many AMP is membrane lipids. AMP interact with anionic phospholipids and phosphate groups of LPS of Gram-negative bacteria and with the peptidoglycan layer made of teichoic and lipoteichoic acids of Gram-positive bacteria (Józefiak and Engberg, 2017). The peptidoglycan architecture of *B. subtilis* differs from other Gram-positive bacteria (Turner et al., 2014; Pasquina-Lemonche et al., 2020). *B. subtilis* has considerably longer glycan strands and a lower degree of peptide crosslinking than *S. aureus*. While *S. aureus* peptidoglycan is dominated by multimeric peptide bonds, *B. subtilis* shows only dimeric linking between peptides (Hayhurst et al., 2008; Vollmer et al., 2008; Vollmer and Seligman, 2010). This difference in bacterial peptidoglycan packing might explain the observed different MIC of anoplin[2-6] toward *B. subtilis* and other Gram-positive bacteria.

The PI uptake assay confirmed that the antibacterial mechanism of anoplin derivatives is related to their destructive effect on cell walls and membrane(s). Namely, anoplin[2-6] effectively passed through the membranes and cell wall of *E. coli* K12 MG1655, while anoplin[5-9] had a stronger destructive effect on *S. aureus* ATCC 29213 cell envelope. These differences occurred probably due to variations in the cell wall structure of Gram-negative and Gram-positive bacteria and variations in the amphipathic helix structure of the two stapled anoplins (Figure 3C). The activity of some antibacterial peptides against Gram-negative bacteria is closely related to their interaction with the outer LPS layer of the cell wall, and the structure they adopt in the proximity to the LPS (Bhunia et al., 2011; Sinha et al., 2017). Thus, we analyzed the relation between the MIC of the peptide against *E. coli* K12 MG1655 and the helicity of the peptide in the presence of LPS, determined from the CD spectra (Supplementary Figure 18). Indeed, we found that the antibacterial activity against *E. coli* is related with the percentage of helicity of the peptide in the LPS. Anoplin[2-6] is the most helical in the presence of LPS and exhibits the highest antibacterial activity. Therefore, the peptide helical content in LPS seems a good determinant of the activities of the peptides against Gram-negative strains.

Anoplin[5-9] has one less positively charged amino acid so is more hydrophobic as compared to anoplin. This change shifts the hydrophobic and hydrophilic parts of the amphipathic helix (Figure 3C). The hydrophobicity of AMP was shown to be a critical element determining the type of cells the peptide is active against. In general, increasing hydrophobicity increases peptide activity against Gram-positive bacteria (and not necessarily against Gram-negative ones; Giangaspero et al., 2001; Wang et al., 2014). The well-described example of how changing hydrophobicity affects AMP activity are for magainin analogs (Wieprecht et al., 1997a,b; Dathe and Wieprecht, 1999). Magainin is a peptide active against Gram-negative bacteria. Increasing its hydrophobicity increased its activity against Gram-positive bacteria and eukaryotic cells (Dathe et al., 1997; Dathe and Wieprecht, 1999). This is also in accord with our observations. Increasing the hydrophobicity of anoplin increased the resulting anoplin[5-9] antibacterial activity, especially toward the Gram-positive strains (Table 1).

Further, our toxicity assays (Figures 3A,B, Table 3; Supplementary Figures 16, 17) and previous works (Chapuis et al., 2012; Wu et al., 2021) support that changing a charged amino acid to S_5_ increases the toxicity of peptide analogs. For peptides with a lower positive charge than the natural anoplin, we observed a dose-dependent reduction in cell viability and increased hemolytic activity. In contrast, anoplin[2-6] was very well tolerated by the cells and RBC. It induced slight changes in cell viability (within 10%) and % hemolysis that were not dose-dependent (Figures 3A,B; Table 3 and Supplementary Figures 16, 17). In agreement with the literature, the cationic charge, the hydrophobic or hydrophilic character, influences the toxicity of α-helical peptides. Those are crucial elements to consider during the design of stapled peptides to increase their antimicrobial potency without a simultaneous increase of their cytotoxicity (Dathe and Wieprecht, 1999).

We found that the stapled anoplins have advantages over unmodified anoplin. We presented peptide modifications that show comparable or even greater antibacterial activity to ampicillin and kanamycin (Table 2). The modifications also improved the proteolytic stability of anoplin. In addition, anoplin[2-6] did not display toxicity to eukaryotic cells, it did not affect their condition or cell membrane continuity. Also, anoplin[2-6] had a comparably low hemolytic activity as anoplin. Stapled peptides have been mostly considered for applications in eukaryotic cells but we believe that stapling can be a promising technique also for antibacterial peptides. We found that stabilization of a helical structure does not necessarily increase the toxicity to eukaryotic cells. This work paves the way for employing other AMP with low activity for secondary structure stabilization by hydrocarbon stapling.

## Data Availability Statement

The original contributions presented in the study are included in the article/Supplementary Material, and further inquiries can be directed to the corresponding authors.

## Author Contributions

MW designed the peptide sequences and performed CD experiments. JMa synthesized the peptides and performed the stability and PI uptake experiments. JMi performed the antibacterial activity assays. RG examined the cytotoxic effect of peptides on eukaryotic cells. JMa, RG, and MW performed the hemolytic assay. MW and JT supervised the project and wrote the manuscript. All authors analyzed and discussed the results, revised the manuscript, and agreed to the published version of the manuscript.

## Funding

We acknowledge funding from the National Science Centre, Poland (SONATA 2019/35/D/NZ1/01957 to MW and JMa and OPUS 2019/33/B/NZ1/01322 to RG).

## Conflict of Interest

The authors declare that the research was conducted in the absence of any commercial or financial relationships that could be construed as a potential conflict of interest.

## Publisher’s Note

All claims expressed in this article are solely those of the authors and do not necessarily represent those of their affiliated organizations, or those of the publisher, the editors and the reviewers. Any product that may be evaluated in this article, or claim that may be made by its manufacturer, is not guaranteed or endorsed by the publisher.
