# Supplementary material for: Stapled Anoplin as an Antibacterial Agent
Source: Front Microbiol. 2021 Dec 13;12:772038. doi: 10.3389/fmicb.2021.772038 (PMC8710804; doi:10.3389/fmicb.2021.772038)
Supplement: Supplementary file 1 [file Data_Sheet_1.pdf]

Stapled anoplin as an antibacterial agent

Monika Wojciechowska[1,*], Julia Macyszyn[1], Joanna Miszkiewicz[1,2], Renata Grzela[1,2], Joanna Trylska[1,*]

[1]Centre of New Technologies, University of Warsaw, Warsaw, Poland

[2]College of Inter-Faculty Individual Studies in Mathematics and Natural Sciences, University of Warsaw, Warsaw, Poland

[3]Division of Biophysics, Institute of Experimental Physics, Faculty of Physics, University of Warsaw, Warsaw, Poland

*** Correspondence:**
Monika Wojciechowska: m.wojciechowska@cent.uw.edu.pl
Joanna Trylska: joanna@cent.uw.edu.pl

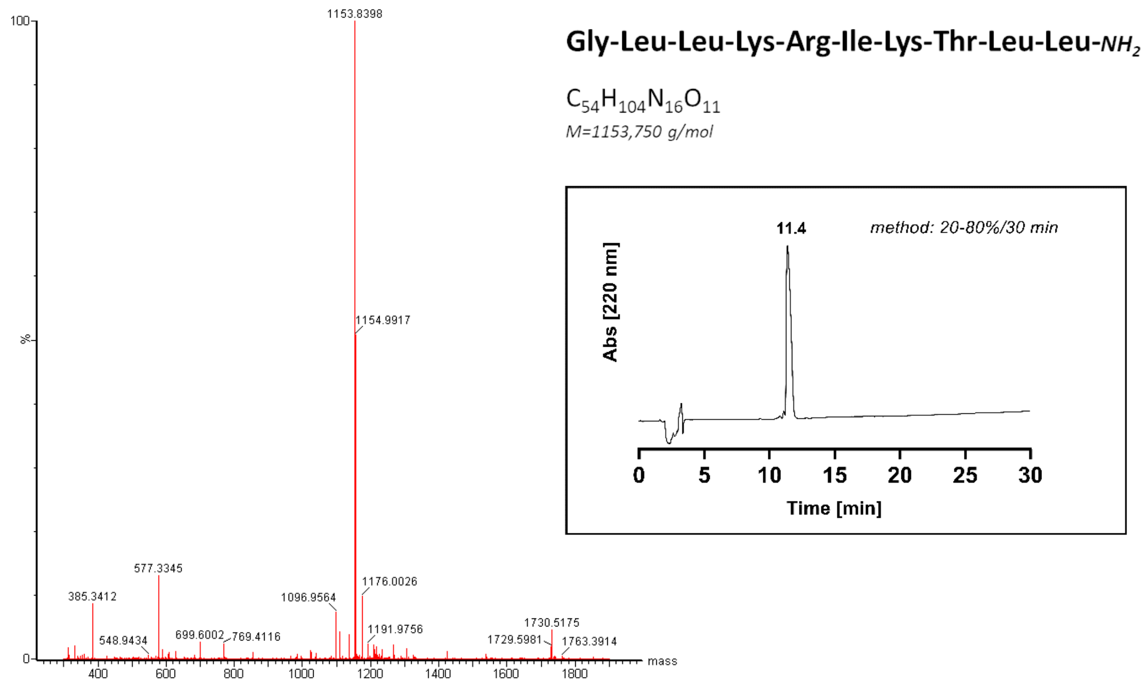

Supplementary Figure 1. The mass spectrum, sequence, chemical formula, calculated mass and RP-HPLC chromatogram of anoplin after purification.

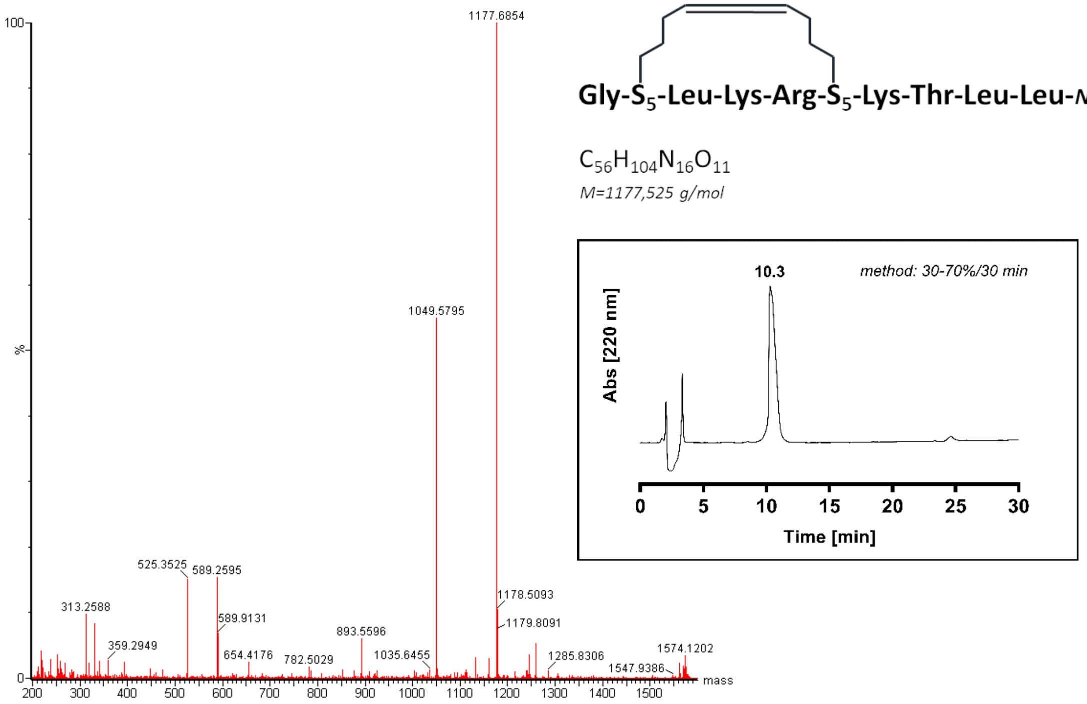

Supplementary Figure 2. The mass spectrum, sequence, chemical formula, calculated mass and RP-HPLC chromatogram of anoplin[2-6] after purification.

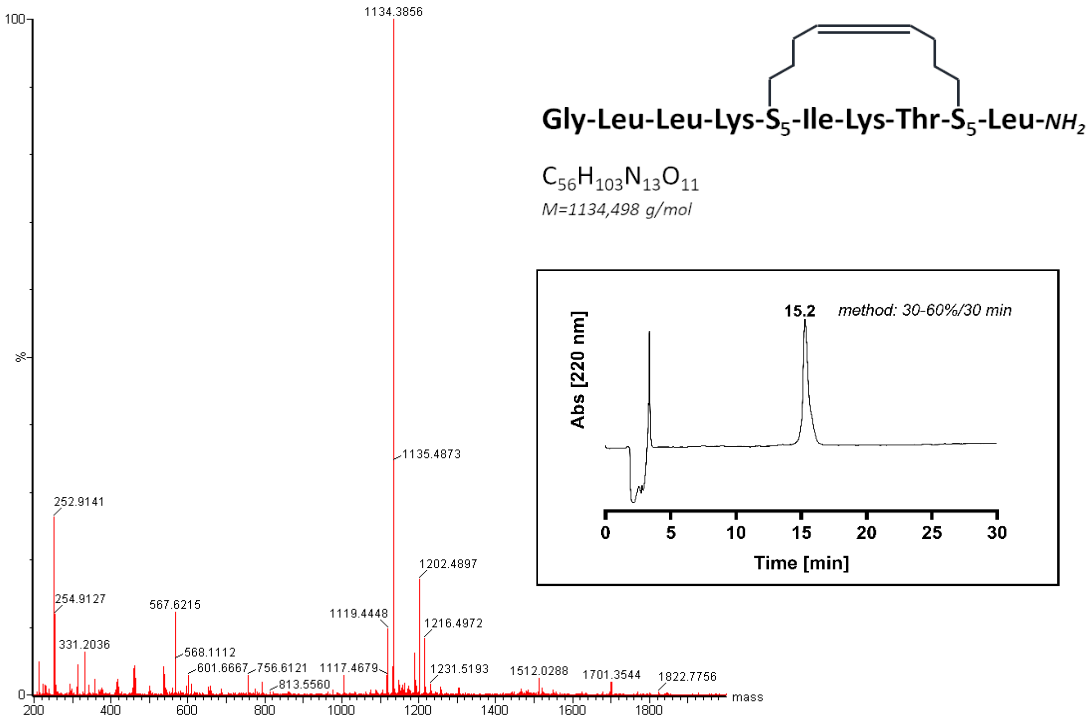

Supplementary Figure 3. The mass spectrum, sequence, chemical formula, calculated mass and RP-HPLC chromatogram of anoplin[5-9] after purification.

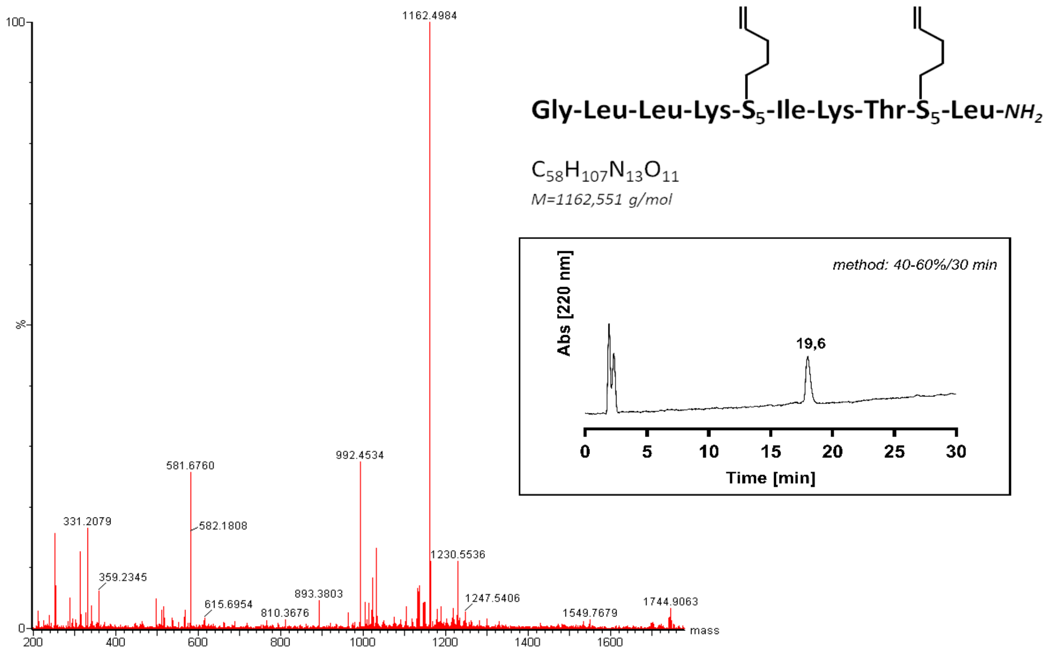

Supplementary Figure 4. The mass spectrum, sequence, chemical formula, calculated mass and RP-HPLC chromatogram of anoplinS_5(5,9) after purification.

Supplementary Table 1. Percentage of helicity calculated from the CD spectra of peptides in different membrane environments: SDS, DPC, POPC:POPG (3:1) SUVs, POPC:POPE (3:1) SUVs and LPS, determined using the DichroWeb software.

Peptide name	helicity [%]					
	buffer	SDS	DPC	POPC:POPG (3:1)	POPC:POPE (3:1)	LPS
anoplin	14	53	55	57	62	56
anoplin[2-6]	59	70	70	63	55	67
anoplin[5-9]	64	71	72	59	59	60
anoplinS_5(5,9)	62	65	75	58	68	54

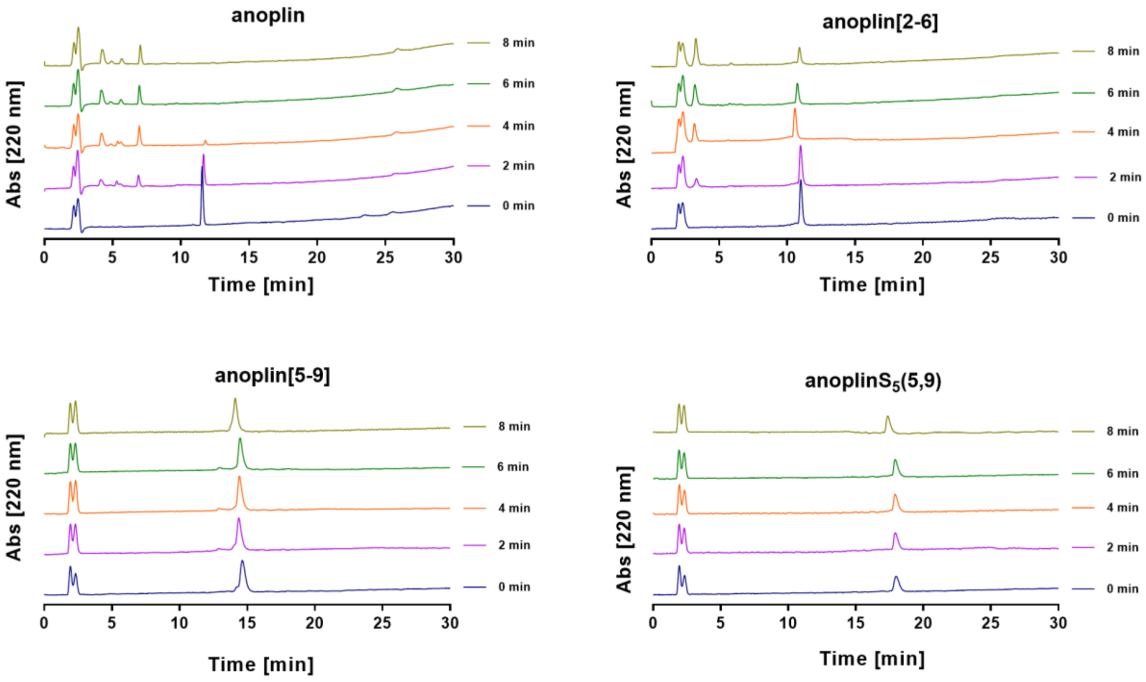

Supplementary Figure 5. The analytical RP-HPLC chromatograms (220 nm) of peptides at different time of incubation with trypsin solution.

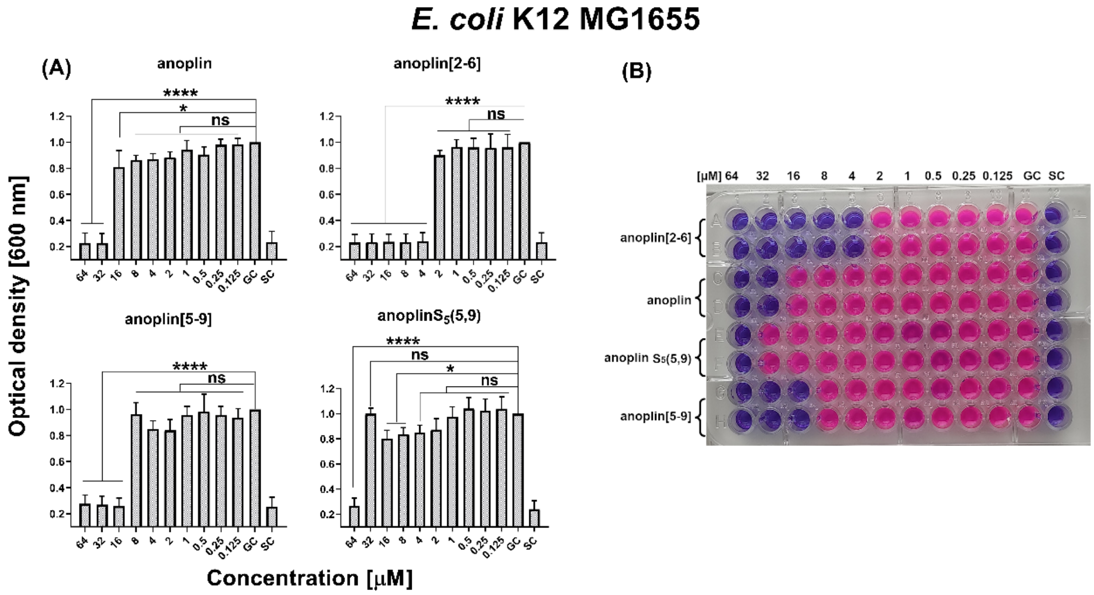

Supplementary Figure 6. The effect of peptide treatment on *E. coli* K-12 MG1655 growth. (A) Optical density shown after 19 h of incubation with various concentrations of anoplin analogs. (B) the resazurin stained plate shown after 1 h of staining. GC – growth control, SC – sterility control. Error bars represent the standard error of the mean; n = 3. Statistical significance between the samples and GC: **** $P < 0.0001$, * $P < 0.05$, ns – not significant.

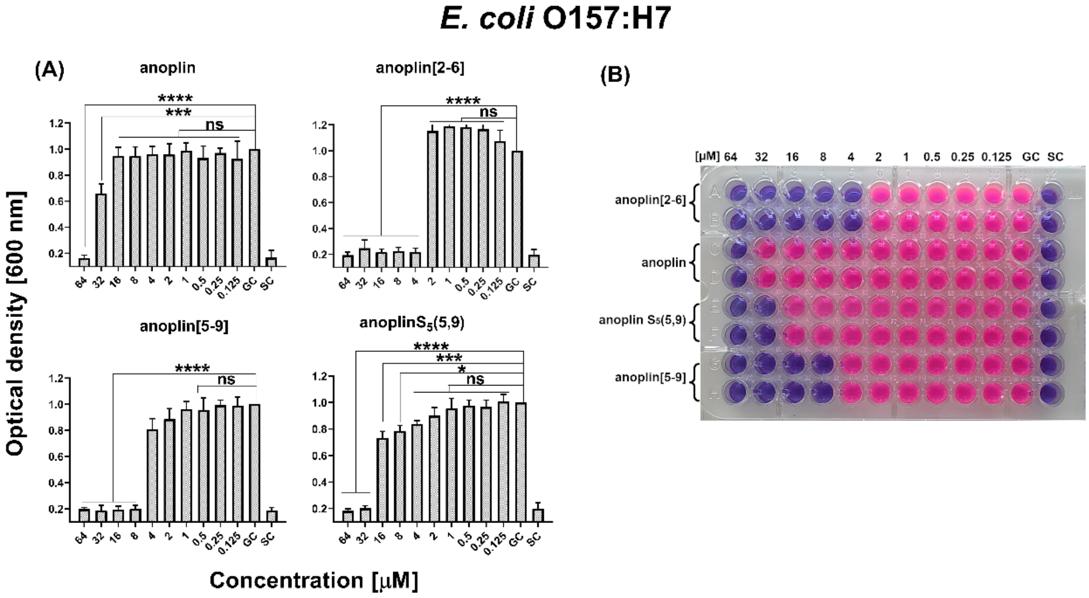

Supplementary Figure 7. The effect of peptide treatment on *E. co*li O157:H7 growth. (A) Optical density shown after 19 h of incubation with various concentrations of anoplin analogs. (B) resazurin stained plate shown after 1 h of staining. GC – growth control, SC – sterility control. Error bars represent the standard error of the mean; n = 3. Statistical significance between the samples and GC: **** P < 0.0001, *** P < 0.001, * P < 0.05, ns – not significant.

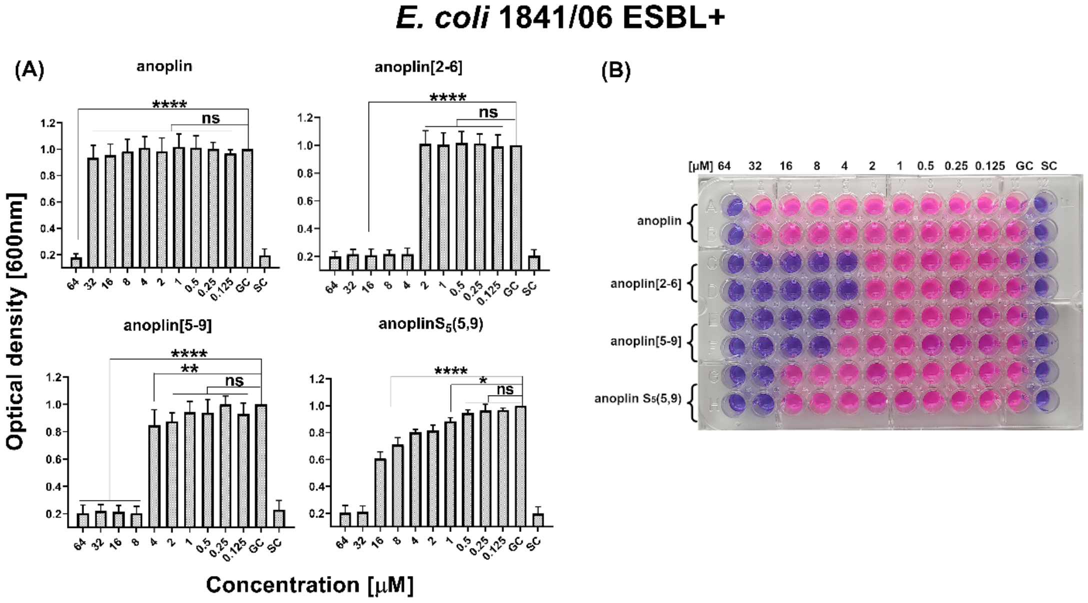

Supplementary Figure 8. The effect of peptide treatment on *E. co*li 1841/06 ESBL+ growth. (A) Optical density shown after 19 h of incubation with various concentrations of anoplin analogs. (B) resazurin stained plate shown after 1 h of staining. GC – growth control, SC – sterility control. Error bars represent the standard error of the mean; n = 3. Statistical significance between the samples and GC: **** P < 0.0001, ** P < 0.01, * P < 0.05, ns – not significant.

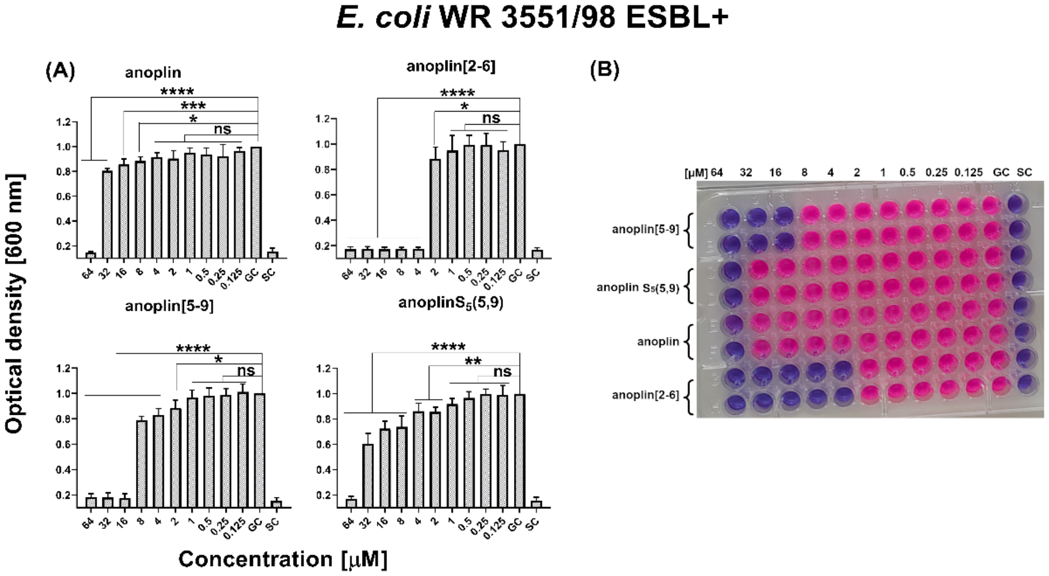

Supplementary Figure 9. The effect of peptide treatment on *E. co*li WR 3551/98 ESBL+ growth. (A) Optical density shown after 19 h of incubation with various concentrations of anoplin analogs. (B) resazurin stained plate shown after 1 h of staining. GC – growth control, SC – sterility control. Error bars represent the standard error of the mean; n = 3. Statistical significance between the samples and GC: **** P < 0.0001, *** P < 0.0001, ** P < 0.01, * P < 0.05, ns – not significant.

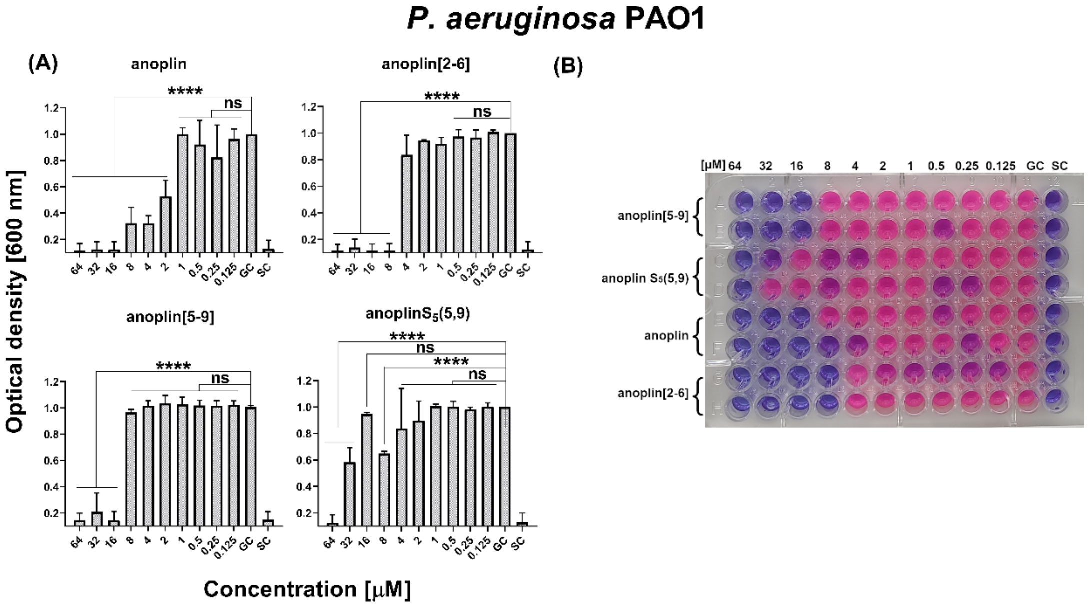

Supplementary Figure 10. The effect of peptide treatment on *P. aeruginosa* PAO1 growth. (A) Optical density shown after 19 h incubation with various concentrations of anoplin analogs. (B) resazurin stained plate shown after 1 h of staining. GC – growth control, SC – sterility control. Error bars represent the standard error of the mean; n = 3. Statistical significance between the samples and GC: **** P < 0.0001, ns – not significant.

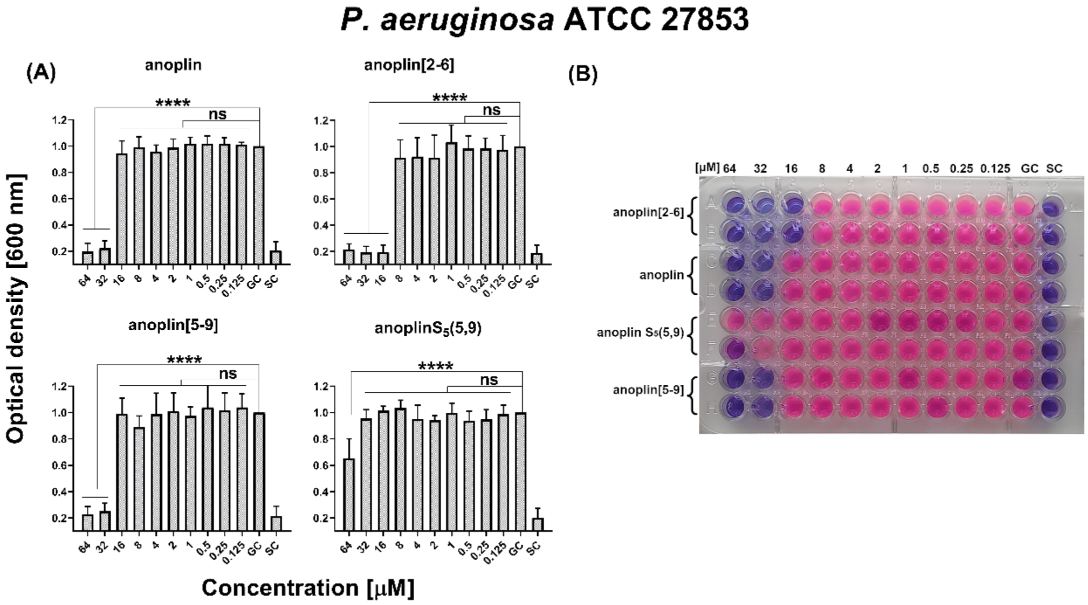

Supplementary Figure 11. The effect of peptide treatment on *P. aeruginosa* ATCC 27853 growth. (A) Optical density shown after 19 h incubation with various concentrations of anoplin analogs. (B) resazurin stained plate shown after 1 h of staining. GC – growth control, SC – sterility control. Error bars represent the standard error of the mean; n = 3. Statistical significance between the samples and GC: **** P < 0.0001, ns – not significant.

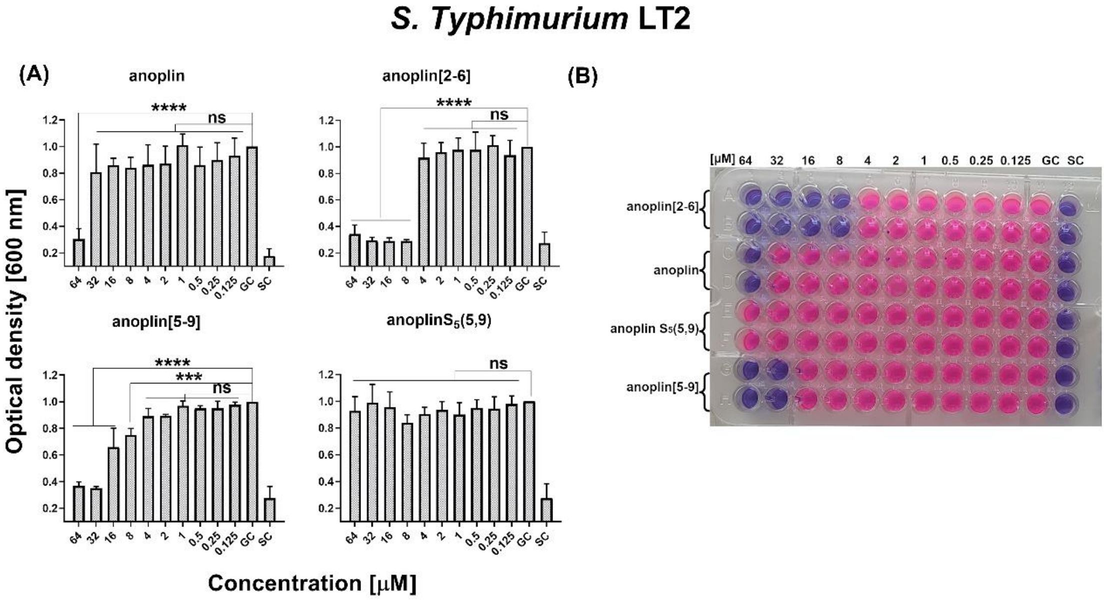

Supplementary Figure 12. The effect of peptide treatment on *S. Typhimurium* LT2 growth. (A) Optical density shown after 19 h incubation with various concentrations of anoplin analogs. (B) resazurin stained plate shown after 1 h of staining. GC – growth control, SC – sterility control. Error bars represent the standard error of the mean; n = 3. Statistical significance between the samples and GC: **** P < 0.0001, *** P < 0.001, ns – not significant.

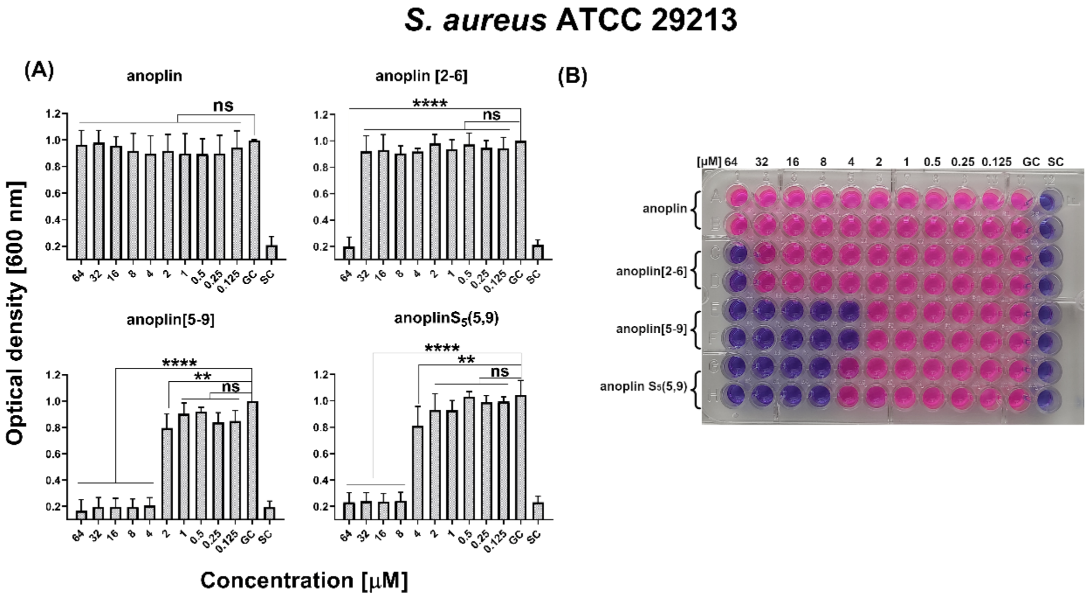

Supplementary Figure 13. The effect of peptide treatment on *S. aureus* ATCC 2913 growth. (A) Optical density shown after 19 h incubation with various concentrations of anoplin analogs. (B) resazurin stained plate shown after 1 h of staining. GC – growth control, SC – sterility control. Error bars represent the standard error of the mean; n = 3. Statistical significance between the samples and GC: **** P < 0.0001, ** P < 0.01, ns – not significant.

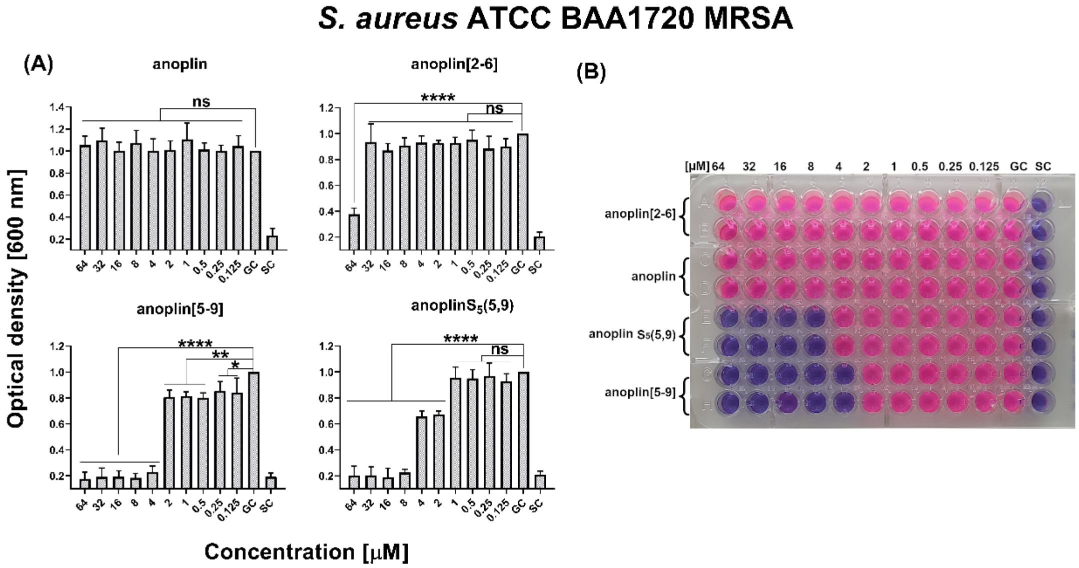

Supplementary Figure 14. The effect of peptide treatment on *S. aureus* ATCC BAA1720 MRSA growth. (A) Optical density shown after 19 h incubation with various concentrations of anoplin analogs. (B) resazurin stained plate shown after 1 h of staining. GC – growth control, SC – sterility control. Error bars represent the standard error of the mean; n = 3. Statistical significance between the samples and GC: **** P < 0.0001, ** P < 0.01, * P < 0.05, ns – not significant.

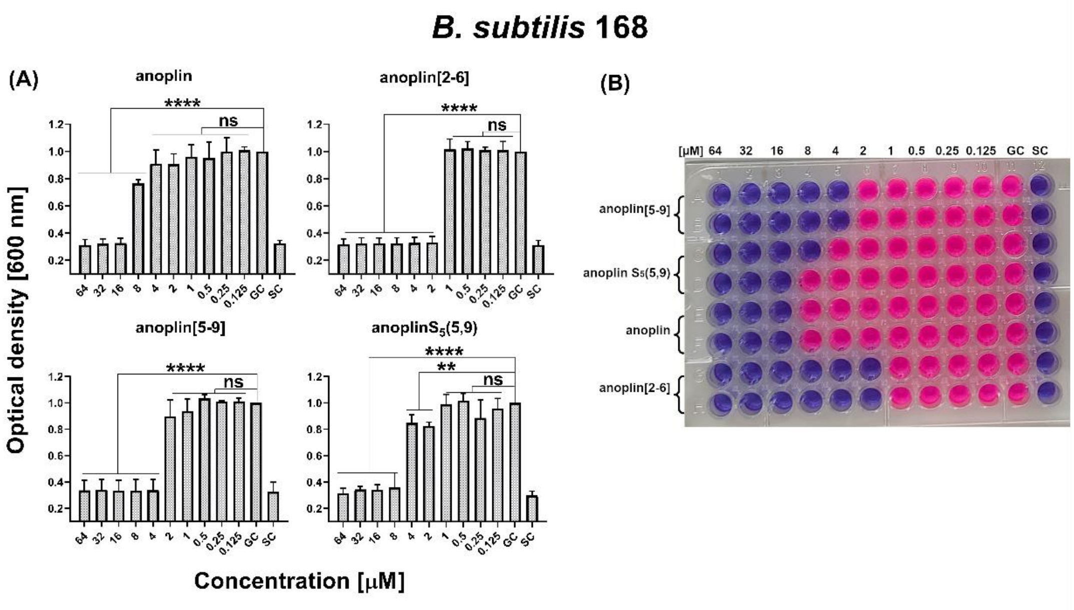

Supplementary Figure 15. The effect of peptide treatment on *B. subtilis* 168 growth. (A) Optical density shown after 19 h incubation with various concentrations of anoplin analogs. (B) resazurin stained plate shown after 1 h of staining. GC – growth control, SC – sterility control. Error bars represent the standard error of the mean; n = 3. Statistical significance between the samples and GC: **** P < 0.0001, ** P < 0.01, ns – not significant.

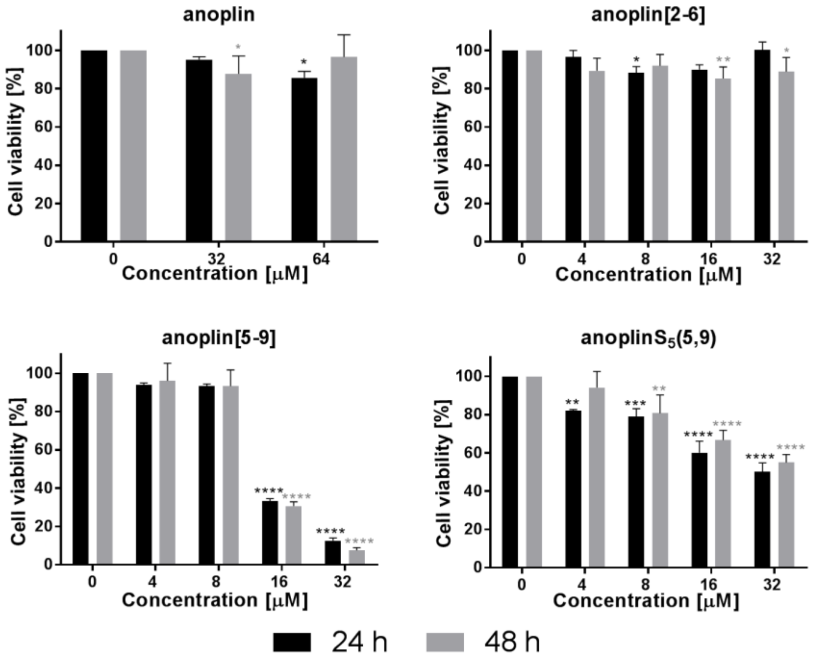

Supplementary Figure 16. The cytotoxic effect of the peptides against the HEK 293 cell line. The results are presented as the % of cell viability in comparison to untreated control cells. Error bars represent the standard error of the mean; n = 3. Statistical significance between the samples and untreated cells: **** P < 0.0001, *** P < 0.001, ** P < 0.01 and * P < 0.05.

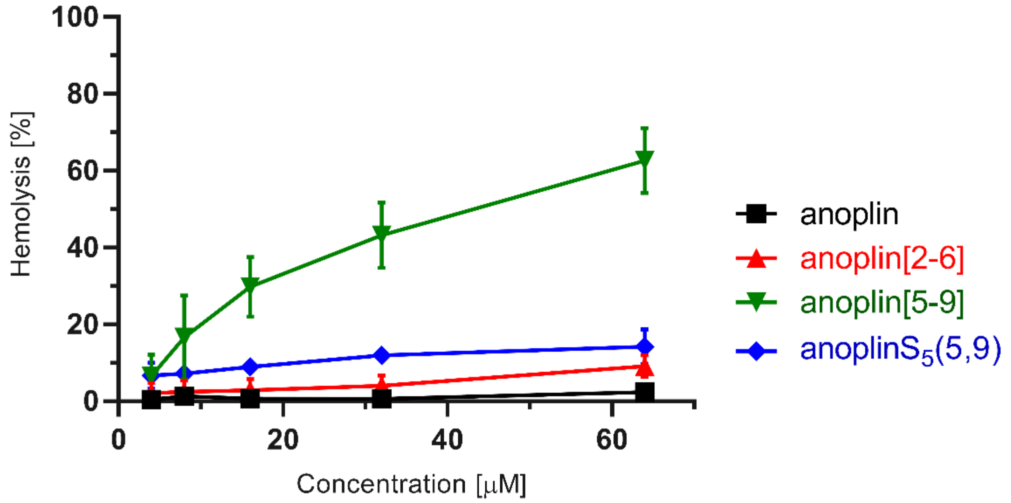

Supplementary Figure 17. Hemolytic activity of anoplin analogs. Sheep erythrocytes were incubated with varying concentrations of the peptide for 30 min. Incubation of erythrocytes with Triton X-100 under the same conditions established 100% hemolysis. Error bars represent the standard error of the mean; n = 3.

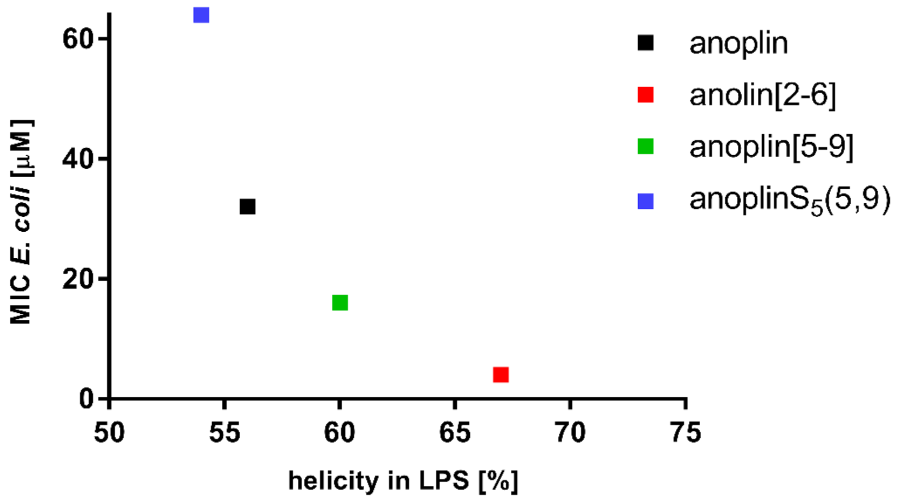

Supplementary Figure 18. The MIC values of the peptides against the *E. Coli* K12 MG1655 strain as a function of the helicity of the peptides in the presence of LPS (determined based on the CD spectra by the DichroWeb serwer).
